# Population, diversity and characteristics of cellulolytic microorganisms from the Indo-Burma Biodiversity hotspot

**DOI:** 10.1186/2193-1801-3-700

**Published:** 2014-11-28

**Authors:** Sailendra Goyari, Shantibala S Devi, Mohan C Kalita, Narayan C Talukdar

**Affiliations:** Institute of Bioresources and Sustainable Development (IBSD), Imphal, India; Gauhati University, Guwahati, India

**Keywords:** Microbial population, Cellulolytic microorganisms, Forest soil, CMC plate assay, FPase activity

## Abstract

Forest ecosystem harbour a large number of biotic components where cellulolytic microorganisms participate actively in the biotransformation of dead and decaying organic matter and soil nutrient cycling. This study explores the aerobic culturable cellulolytic microorganisms in the forest soils of North East India. Soil samples rich in dead and decaying organic matter were collected from eight conserved forests during the season when microbes were found to be most active. Cellulolytic microorganisms were isolated using selective media in which cellulose was the sole carbon source. Population of culturable, aerobic, cellulolytic microorganisms were found to be higher at the incubation temperature that corresponds to the natural ambient temperature of the site of sample collection. Bacterial population was higher in all of the sites than fungal population. Bacterial population ranged from 1.91 × 10^5^ to 3.35 × 10^6^ CFU g^-1^ dry soil while actinomycetes and fungal population ranged from 9.13 × 10^2^ to 3.46 × 10^4^ CFU g^-1^ dry soil and 9.36 × 10^2^ to 4.31 × 10^4^ CFU g^-1^ dry soil, respectively. It was observed that though many isolates showed activity on the CMC plate assay, very few isolates showed significant filter paper activity. Three cellulolytic fungal isolates showing high FPase activity were characterised, identified and submitted to GenBank as *Talaromyces verruculosus* SGMNPf3 (KC937053), *Trichoderma gamsii* SGSPf7 (KC937055) and *Trichoderma atroviride* SGBMf4 (KC937054).

## Introduction

In recent years, due to uncertainty in fossil fuel price and concerns over climate change, there is a global urge in reducing the use of fossil fuels and cut down green-house gas emission. This calls for generation of clean and sustainable energy alternatives. The noble idea of utilizing the biological process to convert cheap and abundant cellulosic biomass to fuels and chemicals offers one of the best choices (Bayer 2009). As of now, microbial conversion is the main route for biomass based alternative fuel production (Alper et al. 2009). Many cellulose degrading microorganisms secrete cocktails of numerous lignocellulolytic enzymes viz. cellobiohydrolase (CBH), *endo*-1, 4-β-D-glucanase (EG) and β-glucosidase (BG) which acts synergistically to degrade lignocellulosic biomass completely (Bisaria et al. 1981). However in the perspective of lignocellulosic based bioethanol production, the costs of these enzymes are still very high (Olson et al. 2011; Klein et al. 2012). Circumventing the high cost of cellulase production remains the top priority in the field of cellulase research. Therefore, there has been much research aimed at obtaining new microorganisms producing cellulolytic enzymes with higher specific activities and greater efficiency (Johnvesly et al. 2002; Lee and Koo 2001; Pattana et al. 2000; Subramaniyan and Prema 2000).

Aerobic microorganisms use different strategies to degrade lignocellulose. They secrete enzymes with different cellulolytic activities. Forest floor and soil horizons harbour ecologically more diverse microbial populations which contribute substantially to decomposition and soil cycling in forest soils (Kellner et al. 2010). In forest ecosystem, cellulolytic fungi are largely responsible for breakdown of large biopolymers viz. cellulose, hemicellulose, lignin and chitin. Fungal cellulase systems consist of a number of exocellulases and endocellulases with different properties and functions in the hydrolysis of crystalline cellulose (Irwin et al. 2000). The ability of microorganisms to degrade cellulose varies not only due to its genetic potential but also due to different environmental conditions within the niche where it operates. North East India represents diverse ecological niches with its unique geography. It stands as the transitional zone between the Indian, Indo-Malayan and Indo-Chinese biogeographic regions and is the geographical gateway for much of India’s flora and fauna (Myers et al. 2000). As a consequence, the area is one of the richest in biological values, high in endemism of biological systems and provides unique niches for the evolution of novel microorganisms. However, the region has remained poorly explored in this aspect. Here, we have attempted to study the population diversity of culturable cellulose degrading microorganisms and characterize efficient cellulolytic fungal isolates from the undisturbed forests of this region.

## Material and methods

### Sample collection

Eight different conserved forest sites located in North East India with different geo-climatic conditions were chosen for sample collection (Table 1). Soil samples were collected from three decomposed organic rich spots of each site where each spot was about 100 ft apart. The subsurface soils from the depth of 0-15 cm were collected in sterile sample containers, wrapped in sterile polythene bag and transported to the laboratory in cool boxes. The samples were then stored in the cold room at 4°C until further analysis.

**Table 1:** **Geographical location of soil sample collection sites**

Name of state	Site of collection	Latitude	Longitude	Altitude (ft)
SIKKIM	Tsomgo Lake Area	N27°22.399	E088°45.500	12400
	Panthang	N27°22.253	E088°35.288	6810
MANIPUR	Mao	N25°31.883	E094°07.946	5680
	Moreh	N24°15.345	E094°17.316	800
ASSAM	Manas National Park	N26°42.864	E090°59.166	523
MEGHALAYA	Shillong Peak	N25°32.539	E091°52.285	6000
AURNACHAL	Namdapha National Park	N27°29.737	E096°23.393	1100
PRADESH	Bomdila	N27°16.343	E092°25.344	8284

### Isolation of cellulolytic microorganisms and population count

Three selective media viz. Omeliansky’s agar, Czapek Dox agar (CDA) and Kenknight and Munier’s agar (K&M) were prepared with slight modification for isolation of bacteria, fungi and actinomycetes, respectively. The carbon source in each of the selective media was substituted with cellulose powder (3 g/L). Five gram of soil sample sieved through 2 mm pore sized sieve was mixed with 45 ml of phosphate buffered saline (PBS). The solution suspension was agitated in a shaker incubator to release the microorganisms from the soil sample. Serial dilution was made in the same buffer. 100 μl of each dilution was pour plated on the modified selective media. The plates were incubated at two temperature (20°C and 30°C) for 3–7 days. All colonies were counted and the bacterial populations were expressed as Colony Forming Unit (CFU) per gram sample dry weight. The population of culturable aerobic microorganisms were determined by plating three replicates of each dilution. Distinct colonies were picked and subcultured to get pure isolates.

### Screening for cellulase activity

Three microliters of bacterial and actinomycetes cultures grown overnight in LB broth media (Himedia) were spot plated on CMC agar (0.2% NaNO_3_, 0.1% K_2_HPO_4_, 0.05% MgSO_4_, 0.05% KCl, 0.2% carboxy methyl cellulose (CMC) sodium salt, 0.02% peptone, and 1.7% agar) (HiMedia, India). A mycelial disc (0.3 cm dia each) of fungal colony was placed on the centre of the modified Cezapak Dox Agar (CDA) plate supplemented with CMC instead of cellulose powder and pH adjusted to 5.3 for screening cellulolytic activity. The culture plates were incubated at 20°C and 30°C. After 4 days of incubation, the culture plates were flooded with Gram’s Iodine for 2-3 min (Kasana et al. 2008). Positive colonies were determined by the relative cellulolytic activity index (RCAI) which compares the diameter of the clearing zone around the colony with the diameter of microbial colony.

### Optimum pH and temperature on growth

Modified CDA plate medium of pH gradient 3.3, 4.3, 5.3, 6.3, 7.3 and 8.3 adjusted with 10% Tartaric acid and 1 M NaOH were prepared. Each set of pH graded media plates were inoculated with one mycelia disc (each 0.3 cm dia) of SGMNPf3, SGBMf4 and SGSPf7 isolates and incubated at 30°C in stationary incubator for 60 hr. The growth in diameter was measured every 12 hr interval. Another set of media plates with pH 5.3 were inoculated with a mycelia disc (each 0.3 cm dia) of the three isolates, each in triplicate and incubated at different temperature range (15°C, 25°C, 30°C and 37°C).

### Optimum pH and temperature on enzyme production

The effect of pH on enzyme production was analysed by varying the pH (3.3 to 8.3) of modified Czapek Dox Broth (CDB) medium supplemented with cellulose powder (3 g/L). Six mycelial discs, each of SGMNPf3, SGBMf4 and SGSPf7 isolates were inoculated in 30 ml CDB medium contained in Erlenmeyer flask and incubated in an orbital shaker (120 rpm) at 30°C for 8 days. Similarly, the effect of temperature was studied at 15°C, 25°C, 30°C, 35°C, 40°C and 45°C, keeping the pH of the culture medium at pH 5.3.

### Enzymes assays

#### Filter paper assay

Filter paper assay was determined by incubating 32 mg of punched pieces of filter paper in 1 ml of 0.05 M Sodium citrate buffer (pH 4.8) with 1 ml of culture supernatant in 50 ml capacity centrifuge tube at 50°C. After 1 hr, 3 ml of Dinitrosalicylic acid (DNS) reagent was added and kept in boiling water bath for 5 min. To stop the reaction, it was kept on ice for 5 min and then 1 ml of 40% Rochelle salt solution was added. A Blank, without filter paper was also run to correct for any reducing sugar present in the enzyme preparation (Eveleigh et al. 2009). One Filter Paper Unit (FPU) was defined as the amount of the enzyme that released 1 μmole of glucose per minute from the original substrate at the experimental conditions.

#### Individual cellulase assays

Endoglucanase and β-glucosidase activity assay were carried out by following the protocol of Thygesen et al. ( 2003). For exoglucanase activity, it was measured by using 1% Avicel (Sigma Chemical, St. Louis, USA) in 100 mM sodium acetate buffer (pH 4.8) as described by Wood and Bhat ( 1988). One unit (IU) of enzyme activity was defined as the amount of the enzyme that released 1 μmole of glucose per minute from the original substrate at the experimental conditions for the endoglucanase and exoglucanase activity assay. For β-glucosidase activity, one unit (IU) is defined as the amount of the enzyme that releases 1 μmole of p- Nitrophenol per minute from the p-Nitrophenyl β-D-glucopyranoside.

#### Protein precipitation

The extracellular proteins from the culture supernatant were precipitated by (NH_4_)_2_SO_4_ at 85% saturation. After standing overnight, the precipitate formed was collected by centrifugation at 10,000 g for 30 min (Thermo Scientific Biofuge Primo R) and then dissolved in 50 mM sodium acetate buffer (pH 5.3). The dissolved sample was dialyzed against the same buffer and concentrated by lyophilisation (Thermo Scientific).

#### Extracellular protein profile

Sodium Dodecylsulfate–Polyacrylamide Gel electrophoresis (SDS–PAGE) was performed according to the method of Laemmli ( 1970) using 12.5% gels. After electrophoresis, the gels were stained by a solution of 0.1% (w/v) Commassie blue, 40% (v/v) methanol and 10% (v/v) acetic acid. The gels were destained by a solution of 40% (v/v) methanol and 10% (v/v) acetic acid and then gels were kept in 7% (v/v) acetic acid. Molecular weight standards (Merck) were used to determine the molecular weight of the extracellular proteins.

#### Genomic DNA isolation

The SGMNPf3, SGBMf4 and SGSPf7 isolates were grown in 30 ml potato-dextrose broth medium for 72 hr at 30°C. The mycelial mat was pelleted by centrifugation (Eppendorf Centrifuge R2050) at 3,000 rpm for 20 min. Genomic DNA was isolated following the rapid extraction method for PCR amplification (Cenis 1992).

#### Identification of three fungi

For sequence analaysis of the ITS1-5.8S-ITS2 r DNA region, PCR was performed in a C1000™ Touch Thermal Cycler (BIORAD, USA) using the primer set: 5'-CTTGGTCATTTAGAGGAAGTAA-3' and 5'-TCCTCCGCTTATTGATATGC-3' according to standard protocol (White et al. 1990). The run was programmed with an initial denaturation at 95°C for 5 min, followed by amplification for 34 cycles at the following conditions: 1 min at 95°C, 1 min at 50°C and 2 min at 72°C. A final 5 min extension at 72°C and infinite hold time at 12°C completed the program run. The amplified products were profiled in 1.5% agarose gel and visualized with ethidium bromide in ChemiDoc™ MP Imaging System (BIORAD, USA). Amplicons of about 450-580 bp were purified and sequenced in ABI370X1 Cycler Sequencer (ABI, USA). The sequences were automatically trimmed and assembled in DNAbaser 3.5.3 software. Following annotation, sequences were assigned to species based on 98-100% sequences similarity threshold in the GenBank. The rDNA sequences of SGMNPf3, SGBMf4 and SGSPf7 were submitted to GenBank® with Accession Numbers KC937053, KC937054 and KC937055, respectively.

#### Statistical significance test

All statistical significance tests were determined by One-way ANOVA using StatPlus. *P < 0.05.

## Result and discussion

### Population count and diversity of cellulolytic microorganisms

**Figure 1:**
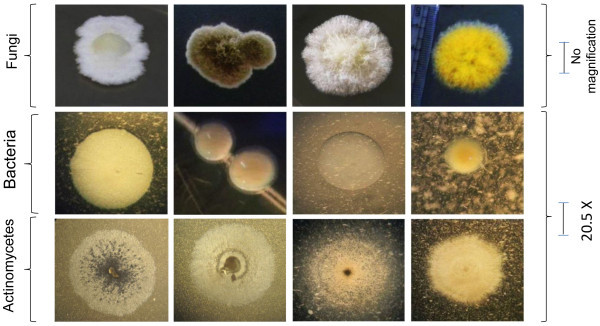
**Colonial morphology of few cellulolytic microorganisms.** Fungal colonies were photographed without any magnification using Nikon D-5000 camera. Bacteria and actinomycetes colonies were photographed using stero zoom microoscope (Olympus) at 20.5X magnification.

Populations of three groups of cellulolytic microorganisms were found to be different in different sites. For example, in the sites like Tsomgo Lake area, Panthang, Mao and Shillong Peak where summer temperature is always below 25°C, the bacterial population ranged from 2.35 × 10^5^ to 3.28 × 10^6^ CFU g^-1^ dry soil and from 1.91 × 10^5^ to 5.2 × 10^5^ CFU g^-1^ dry soil at 20°C and 30°C, respectively. In contrast, the bacterial population in the sites like Moreh, Manas National Park and Namdapha National Park, where summer temperature is always above 25°C, ranged from 1.03 × 10^5^ to 3.08 × 10^5^ CFU g^-1^ dry soil and from 1.91 × 10^5^ to 1.06 × 10^6^ CFU g^-1^ dry soil at 20°C and 30°C, respectively. Similar trend was observed for actinomycetes and fungal population (Table 2). In this study, more microbial colonies were obtained by incubating at the temperature similar to that of the sites from where the samples were collected. This suggests that cellulolytic microorganisms can flourish in culture medium only when they are incubated at their natural ambient environment and therefore, this should be an important consideration in culture based diversity study. It was also observed that the cellulolytic bacterial population was higher compared to actinomycetes and fungi in all of the sites. Among the three groups of microorganisms, fungal population was the lowest. This observation contradicts with many other reports that fungal population dominates bacteria in the forest site with decomposing litter (Baath and Anderson 2003; Baldrian et al. 2012). This contradiction might be due to total soil DNA approach used for estimation of the microbial diversity in their study. However, our results agreed with the report that the ratios of culturable bacteria to culturable fungi were greater in the forest soil, which was based on the classical plate media culturable approach (Matthies et al. 1997). A total of 212 microbial isolates were obtained from the soil samples using cellulose supplemented media (Table 3). The isolates were distinct from one another as evident from their purified colonies on the culture media (Figure 1). On comparison of the colony morphology of the isolates obtained from sample of different sites, it was found that the number of groups of dissimilar colonies was different for different sites. We treated colonies of different isolates obtained from a sample as more diverse, if it had more dissimilar groups of colonies compared to that of another sample. Accordingly, the bacterial diversity was highest in Namdapha National Park and lowest in Tsomgo Lake Area. The actinomycetes diversity was highest in Namdapha National Park and lowest in Tsomgo Lake Area and Manas National Park. Among the different sites, Shillong Peak and Namdapha National Park had more fungal diversity while Moreh had the lowest fungal diversity. It was observed that samples from Namdapha National Park had the highest diversity of the three groups of microorganisms. However, no conclusion could be drawn on the correlation of the diversity of different types of microorganisms and the different temperature regimes of sample sites.

**Table 2:** **Population count of aerobic cellulolyitc microorganisms from different sites**

Site	Annual temperature (°C)	Incubation temperature (°C)	CFU/g dry soil		
			Bacteria	Actinomycetes	Fungi
Tsomgo Lake Area	Max. 10	30	2.10 × 10^5^	8.10 × 10^3^	2.13 × 10^3^
	Min. 2	20	2.34 × 10^5^	3.02 × 10^4^	3.77 × 10^3^
Panthang	Max. 22	30	8.40 × 10^5^	9.13 × 10^2^	9.36 × 10^2^
	Min. 13	20	3.28 × 10^6^	3.46 × 10^4^	6.97 × 10^3^
Manas National Park	Max. 37	30	1.91 × 10^5^	3.06 × 10^3^	3.51 × 10^3^
	Min. 25	20	1.03 × 10^5^	2.11 × 10^3^	2.33 × 10^3^
Mao	Max. 23	30	5.19 × 10^5^	8.79 × 10^3^	3.39 × 10^3^
	Min. 14	20	4.17 × 10^5^	1.61 × 10^4^	1.72 × 10^4^
Moreh	Max. 35	30	1.06 × 10^6^	2.38 × 10^4^	3.98 × 10^3^
	Min. 25	20	3.08 × 10^5^	2.82 × 10^3^	1.69 × 10^3^
Namdapha National Park	Max. 37	30	3.35 × 10^6^	2.74 × 10^4^	9.54 × 10^3^
	Min. 30	20	2.39 × 10^6^	2.20 × 10^4^	4.31 × 10^4^
Bomdila	Max. 26	30	5.2 × 10^5^	8.8 × 10^3^	9.06 × 10^3^
	Min. 11	20	8.5 × 10^5^	9.3 × 10^3^	1.3 × 10^4^
Shillong Peak	Max. 24	30	8.75 × 10^5^	2.98 × 10^4^	6.34 × 10^3^
	Min. 2	20	5.84 × 10^5^	2.15 × 10^4^	1.07 × 10^4^

**Table 3:** **Total number of aerobic cellulolytic microorganisms from soil**

Sample source	Total number of microorganisms		
	Bacteria	Actinomycetes	Fungi
Tsomgo Lake Area	7	2	6
Panthang	8	4	8
Manas National Park	8	2	7
Mao	8	4	6
Moreh	7	4	5
Namdapha National Park	14	25	12
Bomdila	12	13	11
Shillong Peak	13	14	12
Total	77	68	67

### Qualitative and quantitative cellulase activity

Of the 212 isolates, 168 showed CMC hydrolysis zone in the plate assay. The RCAI values of bacteria and actinomycetes ranged from 0.8 to 2.3 cm whereas for fungi, it ranged from 0.4 to 2.6 cm. The FPase activity of bacteria and actinomycetes ranged from 0.3 to 8 FPU whereas for fungi, it ranged from 2 to 44 FPU. It was observed that high RCAI index on CMC plate assay did not correspond to high FPase activity for bacteria and actinomycetes (Table 4). Dashtban et al. ( 2010) reported that Gram’s iodine was superior test than others for a fast and easy detection of endoglucanase activity on CMC plate assay. Their results clearly indicated that those organisms which had less or no endoglucanase activity did not show the hydrolysis zone on the CMC plate assay. This might suggest that the bacterial and actinomycetes isolates did not produce the other cellulase enzymes whereas SGMNPf3 produced all and hence, showed positive in both the assays. Thus, it infers that CMC plate assay is not sufficient to conclude the cellulase producing potential of microorganisms.

**Table 4:** **Comparative analysis on cellulolytic activities of different microbial isolates**

Microorganism	Isolate code	RCAI (cm)	FPase (IU/L)
Fungi	MHf2	1.5 ± 0.02	13 ± 1.40
	MOf5	1.1 ± 0.03	4.5 ± 0.13
	MNPf3	2.6 ± 0.01	44 ± 2.30
	BMf4	1.6 ± 0.03	21 ± 1.33
	SPf7	0.9 ± 0.04	30 ± 2.11
	*Activity range*	0.4-2.6	2-44
Bacteria	MHb5	1.2 ± 0.02	1.8 ± 0.20
	MOb1	1.4 ± 0.03	1.2 ± 0.30
	MNPb5	2.0 ± 0.05	2.1 ± 0.40
	NNPb11	0.8 ± 0.02	1.1 ± 0.10
	SLb3	1.2 ± 0.02	1.5 ± 0.60
	*Activity range*	0.8-2	0.4-2
Actinomycetes	SPac10	2.3 ± 0.08	6.7 ± 1.40
	NNPac3	2.1 ± 0.04	1.4 ± 0.10
	MHac7	1.1 ± 0.05	4.1 ± 0.30
	SLac1	1.6 ± 0.01	0.8 ± 0.01
	MHac5	2.2 ± 0.02	8.1 ± 2.11
	*Activity range*	0.8-2.3	0.3-8

### Identification of three fungal isolates

**Figure 2:**
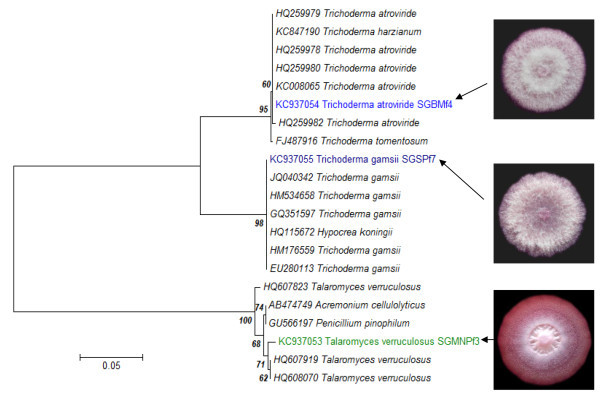
**The phylogenetic dendrogram for** ***T. atroviride*** **SGBMf4,** ***T. gamsii*** **SGSPf7 and** ***T. verruculosus*** **SGMNPf3 and related strains based on the ITS rDNA sequence.** Numbers following the names of the strains are accession numbers of published sequences.

Colonial morphology of SGBMf4 and SGSPf7 were spreading and white floccose mycelia. The mycelia of SGSPf7 were covered with greenish spores after 10 days of incubation while mycelia of SGBMf4 were observed to be covered with green spores in the centre and yellowish towards the margin. The elevation of mycelial colonies of SGMNPf3 were flat on the edge, slightly floccose at the centre and sporulation was indistinguishable. The ITS sequences of the three potential fungal isolates viz. SGMNPf3, SGBMf4 and SGSPf7 were submitted to GenBank with accession numbers- KC937053, KC937054 and KC937055, respectively. The isolate SGMNPf3 showed highest identity to *Talaromyces verruculosus* (99.1%), SGBMf4 to *Trichoderma atroviride* (99.5%) and SGSPf7 to *Trichoderma gamsii* (99.9%) in the NCBI blast search. Phylogenetic relationships were drawn using alignment and cladistics analyses of homologous nucleotide sequences of known microorganisms (Figure 2). Based on their colonial morphology and comparisons of their ITS rDNA gene sequences, the isolated strains were identified and named as *Talaromyces verruculosus* SGMNPf3, *Trichoderma atroviride* SGBMf4 and *Trichoderma gamsii* SGSPf7.

### Growth and cellulase production parameters of three fungal isolates

**Figure 3:**
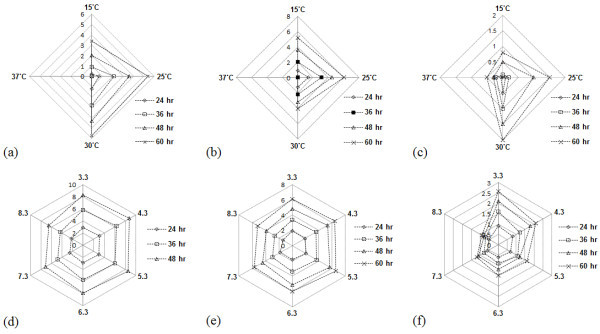
**Growth pattern of fungal isolates at varying pH and temperature.** Growth pattern of SGSPf7 at different pH **(a)** and temperature **(d)**; Growth pattern of SGBMf4 at different pH **(b)** and temperature **(e)**; Growth pattern of SGMNPf3 at different pH **(c)** and temperature **(f)**.

**Figure 4:**
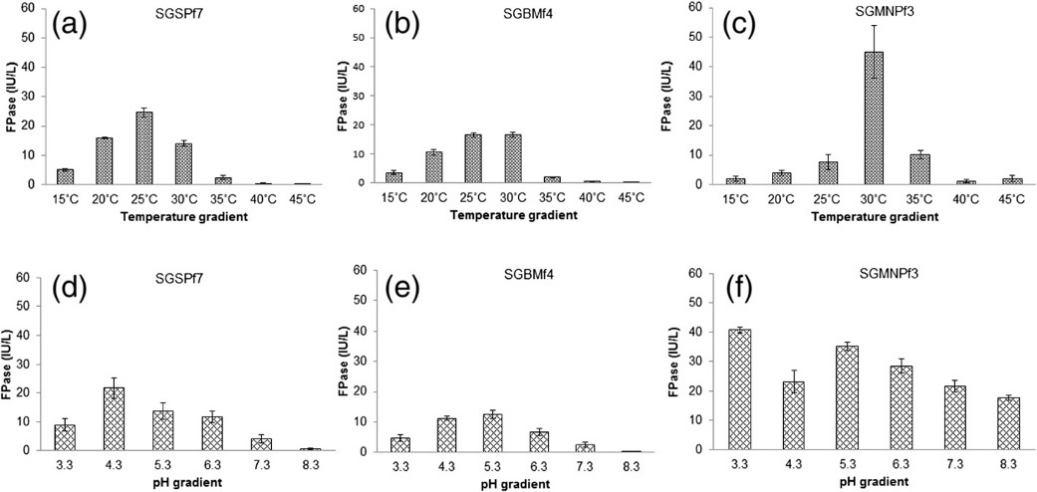
**Cellulase production pattern of fungal isolates at varying pH and temperature.** Pattern of SGSPf7 at different pH **(a)** and temperature **(d)**; Pattern of SGBMf4 at different pH **(b)** and temperature **(e)**; Pattern of SGMNPf3 at different pH **(c)** and temperature **(f)**.

Temperature and pH are the important physiological parameters affecting the growth and enzyme production of microorganisms. In our study, optimum growth rate of *T. atroviride* SGBMf4, *T. gamsii* SGSPf7 and *T. verruculosus* SGMNPf3 were observed at pH 5.3, 4.3 and 3.3 (Figure 3a, b & c) and at temperature 25°C, 25°C and 30°C (Figure 3d, e & f), respectively. This observation reflects well to the temperature condition of sample collection sites. *T. atroviride* SGBMf4 had higher growth rate and *T. verruculosus* SGMNPf3 was the slowest. The result agreed with the reports that *Trichoderma* spp were fast growing at 25-30°C (Gams and Biset 1998; Yu et al. 2014). It was observed that *Trichoderma* isolates did not grow above 30°C except *T. verruculosus* SGMNPf3, which was able to grow at 37°C. The pH and temperature at which the growth of the three fungi were optimum, were also observed to be the favourable condition for maximum enzyme production. Highest cellulase production of SGSPf7 (25 U/L) was observed at 25°C and pH 4.3 (Figure 4a & d), SGBMf4 at 25°C and pH 5.3 (Figure 4b & e) and SGMNPf3 (40 IU/L) at 30°C and pH 3.3 (Figure 4c & f). Similar observations were also reported by Singh et al. ( 2014). Though SGMNPf3 was a slow grower, it surpassed the other isolates in cellulase production potential. The acidophilic characteristic of SGMNPf3 makes it a promising candidate for industrial purposes since most of the substrates are pre-treated with inorganic acids (Hahn-Hagerdal et al. 2006; Tavares et al. 2013).

### Individual cellulase activity

**Figure 5:**
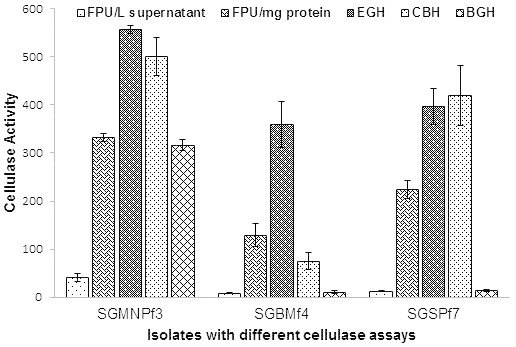
**Individual cellulase production of three potential cellulolytic fungus.**

The three fungal isolates viz. *T. verruculosus* SGMNPf3, *T. gambii* SGSPf7 and *T. atroviride* SGBMf4 showed all of the three individual cellulase activities (Figure 5). The endoglucanase activity was observed to be highest for SGMNPf3 (555.8 IU/mg) and SGBMf4 (358.9 IU/mg) whereas exoglucanase activity was found to be highest for SGSPf7 (419.3 IU/mg) in their individual cellulase assays. The β-glucosidase activity was very low for SGBMf4 (10.3 IU/mg) and SGSPf7 (14.1 IU/mg) whereas it was significantly high in SGMNPf3 (316.1 IU/mg). It has been reported that fungi belonging to genus *Trichoderma*, produce low amount of β-glucosidase (Gritzali and Brown 1979; Ma et al. 2011) while those that belong to genus *Penicillium* are good producers (van Wyk 1999; Castellanos et al. 1995; Jorgensen et al. 2005). This report strongly supports our observations. In this study, *T. verruculosus* SGMNPf3 showed higher individual cellulase activities compared to *T. gambii* SGBMf4 and *T. atroviride* SGSPf7. Though genus *Trichoderma* has been reported (Herpoel-Gimbert et al. 2008) to be high cellulase producer, *Talaromyces* which is a teleomorphic state of genus *Penicillium* had shown to be a better cellulase producer.

### Extracellular protein profile

**Figure 6:**
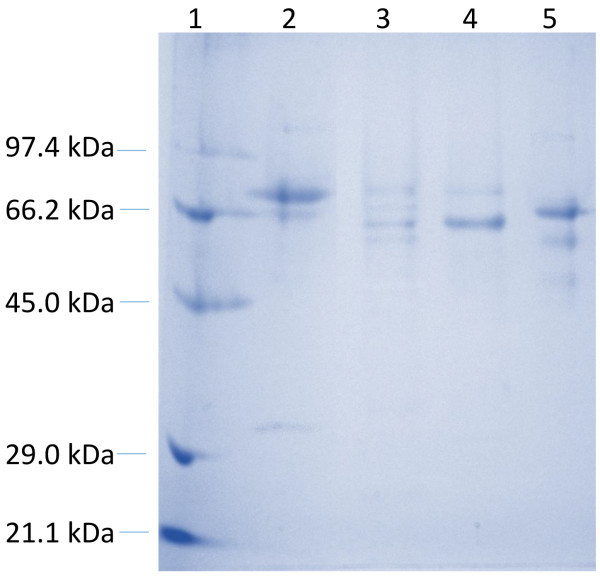
**Extracellular protein profile of three cellulolytic fungus.** Lane 1: Molecular Weight Marker; lane 2: SGMNPf3 sample; lane 3: SGSPf7 sample; lane 4 SGBMf4; lane 5: Sigma cellulase.

Understanding the different enzymes or protein secretions of microorganisms would unveil the mechanisms they adopts in the process of biomass degradation. The three fungal isolates revealed different extracellular protein secretions when grown on cellulose substrates. All the three fungi showed dominant extracellular protein bands which were in the molecular weight range of 60‒75 kDa (Figure 6). *T. verruculosus* SGMNPf3 and *T. gamsii* SGBMf4 showed two protein bands while *T. atroviride* SGSPf7 showed three protein bands which had molecular weight in the range of commercial cellulases (Sigma Aldrich) from *Trichoderma reesei*. A protein band of 36.5 kDa was observed in the extracellular protein sample of *T. verruculosus* SGMNPf3 which was not observed in the other isolates and commercial cellulase.

## Conclusion

In the biomass degradation viewpoint, the population density of culturable cellulolytic bacteria was found to be higher compared to the fungi in the forest soil. Microorganisms seems to thrive well in their native ambient pH and temperature. Although many microorganisms show cellulolytic activity, very few cellulolytic microorganisms have the potential to attract industrial application. Hence in this regard, cellulolytic potential of *T. verruculosus* SGMNPf3, *T. gamsii* SGSPf7 and *T. atroviride* SGBMf4 isolated from the Indo-Burma Biodiversity hotspot need to be further explored for activity of their purified cellulases and possible commercial scale cellulase production in future.
